# Differentiated Empowerment and Boundary Effects of AI-Assisted Music Learning: A Mixed-Methods Study of Learning Motivation, Self-Regulated Learning, and Creative Performance

**DOI:** 10.3390/jintelligence14070126

**Published:** 2026-07-01

**Authors:** Minbo Li, Yunyi Zhao, Xin Shan, Xiaofei Du

**Affiliations:** 1 College of Music, Yunnan Arts University, Kunming 650504, China; 2 The Graduate School Arts and Culture, Sangmyung University, Seoul 03016, Republic of Korea; 3 College of Mechanical and Electrical Engineering, Harbin Engineering University, Harbin 150001, China

**Keywords:** artificial intelligence assistance, boundary conditions, music education, motivation, self-regulated learning, creativity, higher-order cognition, three-level meta-analysis

## Abstract

Although artificial intelligence (AI) is reshaping music education, outcome-oriented quantitative syntheses remain relatively limited. This mixed-methods review examined the effects of AI-assisted music learning on learning motivation, self-regulated learning (SRL), and creative performance, while identifying learner-, task-, and time-related boundary conditions and clarifying how AI support is implemented and experienced. A three-level meta-analysis was used for quantitative integration, complemented by a qualitative synthesis of implementation pathways and learner experiences. Results showed positive trends across all three core domains, with different levels of statistical support and substantial heterogeneity. Learning motivation showed the most robust evidence (*g* = 1.28), creative performance showed a larger but highly heterogeneous effect (*g* = 1.21), and SRL showed a preliminary positive trend (*g* = 0.57). The task complexity × prior ability interaction provided tentative, directional evidence for learner–task fit, mainly for motivational outcomes. Dose-related analyses suggested a possible asynchronous pattern: motivational gains may emerge rapidly in the short term, whereas gains in higher-order cognition may become more evident under sustained intervention. Qualitative synthesis identified three AI implementation pathways—evaluative feedback, generative support, and adaptive personalization—suggesting that effectiveness depends less on technological complexity itself than on aligning AI roles with task demands and learner needs. Future research should strengthen long-term designs, deepen SRL-related evidence, and examine the adaptive effects of different AI roles across diverse music learning contexts.

## 1. Introduction

Against the backdrop of digitalized education and the rapid development of generative artificial intelligence, artificial intelligence (AI) has become a major topic in educational technology research and has gradually expanded into such areas of music learning as practice, feedback, assessment, and creative production (Cheng, 2025; Xia et al., 2025).

However, despite continuous technological advancement, improvements in the quality of music learning still depend to a considerable extent on the coordinated development of learners’ internal systems. More specifically, learning motivation, self-regulated learning (SRL), and creative performance may be regarded as three interrelated core dimensions of music learning, corresponding respectively to the motivational basis of learning engagement, the regulatory mechanisms operating throughout the learning process, and higher-level learning outcomes. Importantly, SRL and creativity not only reflect learners’ higher-order cognitive activity at the individual level, but may also be understood as important manifestations of abilities closely related to intelligence (Karwowski et al., 2016; Sternberg, 1999; Veenman et al., 2004). SRL, which relies on metacognitive monitoring, strategy selection, and adaptive regulation, is closely associated with intellectual development and plays an important role in complex learning tasks (Veenman et al., 2004; Y. Wang et al., 2025; Zimmerman, 2002). Creativity, by contrast, refers to the ability to generate ideas or products that are both novel and valuable; within the theory of successful intelligence, it is regarded as an important component of the broader intelligence system, and empirical research has shown that it is stably associated with intellectual performance (Karwowski et al., 2016; Runco & Jaeger, 2012; Sternberg, 1999). From this perspective, examining how AI is associated with this coordinated system—particularly the ways in which it may support or interfere with higher-order cognition and intelligence-related activity—is essential for understanding the educational value of AI-assisted learning.

In recent years, however, review studies have mainly focused on application scenarios and pedagogical integration in AI-supported music education (Cheng, 2025; Mazlan et al., 2026), the classification of tools and technical systems (A. Dash & Agres, 2024; Kyriakou et al., 2024), and such issues as instructional challenges, ethics, and future directions (Cheng, 2025; Fang, 2026; Mazlan et al., 2026). By contrast, evidence synthesis on deeper learner outcomes remains relatively limited, especially with respect to the systematic quantitative integration of three core outcomes: learning motivation, SRL, and creative performance. More importantly, existing research still lacks sufficiently integrated evidence regarding the boundary conditions of AI effects across different learners, task requirements, and intervention durations, as well as the mechanisms through which such effects are implemented. In other words, the educational significance of AI-assisted learning lies not only in whether it shows positive effects on average, but also in whether such effects can be interpreted in a relatively stable and meaningful way across different conditions. Without closer attention to these issues, our understanding of heterogeneous findings remains constrained, and the practical value of the research for precision teaching and differentiated support is weakened.

At the methodological level, conventional meta-analysis also has limitations in addressing the complex forms of heterogeneity involved here. To evaluate this “motivation–process–outcome” system more comprehensively, many empirical studies simultaneously measure and report multiple related outcomes—such as motivation, SRL, and creativity—within the same independent sample, thereby violating the assumption of effect-size independence in traditional meta-analysis. To address this issue, the present study adopts a three-level meta-analysis, which makes it possible to model the nesting and dependence of multiple effect sizes within studies and, on this basis, to examine more complex moderation effects, such as the interaction between task cognitive complexity and prior ability. At the same time, because quantitative models alone are still insufficient to fully capture the concrete implementation of AI support and the lived experiences of learners, this study further incorporates a qualitative synthesis to complement the contextual and mechanistic insights underlying the statistical findings.

In sum, existing reviews have provided valuable insights into AI tools, application scenarios, pedagogical integration, and ethical challenges in music education. However, less is known about how AI-assisted music learning is associated with deeper learner outcomes, how these associations vary across learner-, task-, and time-related conditions, and how different forms of AI support are implemented in specific educational contexts.

## 2. Theoretical Framework

### 2.1. Internal Driving Mechanisms of the Main Effects

AI-assisted learning may produce baseline main effects through a dual pathway of psychological activation and cognitive scaffolding. According to self-determination theory (SDT), learners’ intrinsic motivation is more likely to be activated when their needs for autonomy and competence are supported (Deci & Ryan, 2000). In music learning, the immediate feedback, low-risk trial-and-error space, personalized suggestions, and generative support offered by AI may enhance learners’ sense of control and competence, thereby increasing engagement and willingness to persist in practice (Cheng, 2025; Ou et al., 2025). At the same time, SRL theory emphasizes that complex music learning depends on learners’ active regulation during practice, monitoring, correction, and reflection (Zimmerman, 2002). In this process, AI may function as an external scaffold by supporting feedback acquisition, error detection, process monitoring, and structured reflection, thereby providing external support for learners’ planning, evaluation, and adjustment (W. Li et al., 2025; Zimmerman, 2002).

However, such support does not necessarily translate directly into immediate or uniform improvements in SRL, because the development of regulatory ability depends more strongly on learners’ active appropriation and sustained use of strategies than on the mere presence of external support itself (Mieder & Bugos, 2017; Zimmerman, 2002). By contrast, when AI lowers barriers to task entry, strengthens learning engagement, and expands the space for idea generation, creative performance is more likely to be activated within a shorter period of time; however, such gains still depend on whether learners use AI support in an active, critical, and iterative manner (Xia et al., 2025; Baltà-Salvador et al., 2026). This interpretation is also consistent with prior creativity research showing that motivational orientation can shape ideational flexibility and thereby further influence the quality of creative thinking (Y. Li et al., 2019).

Based on this logic, the present study proposes that AI-assisted learning may show an overall positive tendency across learning motivation, SRL, and creative performance, although the magnitude, robustness, and underlying mechanisms of these effects may differ across outcome domains.

### 2.2. Learner and Task Boundaries

From the perspective of cognitive load theory (CLT), the value of instructional support lies not in simply adding information, but in helping learners reduce unnecessary extraneous load and preserve limited cognitive resources for schema construction and complex processing (Sweller, 1988; van Merriënboer & Sweller, 2005). Furthermore, the expertise reversal effect suggests that the same external scaffold may support learners with low prior ability but become redundant for those with high prior ability; when scaffolds are mismatched with existing schemas, deep processing may be inhibited rather than facilitated (Kalyuga et al., 2003). Recent theoretical integrations have also suggested that cognitive load not only affects information processing but may also generate motivational costs that reduce learners’ willingness and capacity to invest in a task (Evans et al., 2024; Feldon et al., 2019).

In AI-assisted music learning contexts, this implies that the effect of AI support may not be determined by learners’ prior ability or task cognitive complexity in isolation, but rather by the degree of fit between them. In other words, the effectiveness of AI intervention may be subject to clear learner and task boundaries: when scaffold strength, task demands, and learners’ existing ability levels are appropriately matched, AI is more likely to function supportively; when they are mismatched, its facilitating effects may weaken or even result in cognitive redundancy or processing interference.

### 2.3. Theoretical Temporal Boundaries

Early educational technology research suggested that when learners first encounter a new medium, its novelty may produce short-term gains in motivation and engagement; however, such initial advantages do not necessarily translate into stable learning gains (Clark, 1983). Later longitudinal studies similarly showed that the engagement benefits brought by technological tools may be strongest at the beginning, while this “novelty advantage” may diminish as learners become more familiar with the tool, thereby requiring deeper forms of instructional support to sustain continued effects (Jeno et al., 2019; Tsay et al., 2020).

In AI-assisted music learning, positive effects at low doses may therefore appear mainly as heightened interest, novelty-driven feedback salience, or short-term gains in self-efficacy. By contrast, higher-level outcomes such as SRL and creative performance are typically more cumulative and lagged: The former depends on sustained cycles of monitoring and reflection (Zimmerman, 2002), whereas the latter requires longer periods of practice, strategy internalization, and the integration of generative processing. This is also consistent with broader creativity research suggesting that real-life creativity often depends on deeper internal cognitive pathways (Y. Li et al., 2022). Consequently, different outcome variables may not develop synchronously under AI intervention, but may instead follow a staged process in which motivational activation appears first and higher-order cognitive gains become progressively externalized over time.

From this temporal perspective, the present study examines whether observed outcome patterns vary by intervention dose, but does not assume a simple linear accumulation of benefits. Rather, different learning outcomes may follow distinct developmental trajectories. In particular, motivational outcomes may be more likely to emerge in the early stages of intervention, whereas higher-order outcomes such as SRL and creative performance may become more evident only over longer time spans.

### 2.4. Positioning of the Present Study and Research Questions

Music learning is not merely a cognitive task, but involves the coordination of cognitive processing, affective engagement, bodily action, auditory–motor adjustment, and subjective aesthetic judgment. This ecological specificity means that AI-assisted music learning should be examined not only in terms of average effects, but also in terms of how AI support is embedded in concrete musical action and human judgment.

Building on the preceding theoretical discussions of psychological activation, cognitive scaffolding, learner–task fit, and temporal boundaries, the core purpose of this study is to address the current evidence gap in AI-assisted music learning regarding outcome-oriented quantitative integration, differentiated boundary testing, and the linked interpretation of implementation mechanisms. Specifically, by combining a three-level meta-analysis with a qualitative synthesis, this study examines the average intervention effects and evidential robustness of AI-assisted music learning on learning motivation, SRL, and creative performance, while further exploring whether these outcomes vary according to learner conditions, task complexity, and intervention duration.

Given the substantial heterogeneity across educational levels, task types, AI tools, and measurement approaches, as well as the ecological complexity of music learning, this study is framed as an exploratory evidence synthesis. Effect estimates are therefore interpreted as context-sensitive patterns rather than as universally generalizable causal estimates. This positioning allows the study to map the current evidence landscape, identify potential boundary conditions, and clarify how different forms of AI support are implemented and experienced in specific educational settings. Accordingly, this study addresses the following four exploratory research questions (RQ):

RQ1: What effects of AI-assisted music learning are observed on learners’ motivation, SRL, and creative performance?

RQ2: Do task cognitive complexity and learners’ prior ability interactively moderate the outcomes of AI-assisted music learning?

RQ3: Does intervention dose moderate the effects of AI-assisted music learning?

RQ4: In AI-assisted music learning contexts, how is AI support for learners’ motivation, SRL, and creative performance concretely implemented and experienced, and what common patterns and key differences emerge?

## 3. Methods

### 3.1. Literature Search and Screening Strategy

This study adopted a mixed systematic review design that combined three-level meta-analysis with qualitative synthesis, and literature identification, screening, and reporting were conducted in accordance with the PRISMA 2020 statement (Page et al., 2021). The systematic literature search was completed on 27 March 2026 and aimed to identify, as comprehensively as possible, empirical studies examining the effects of AI-assisted music learning on learning motivation, SRL, and creative performance. The study protocol was not prospectively registered. However, to enhance transparency and reproducibility, complete records of the search strategies, eligibility criteria, screening decisions, extracted effect sizes, moderator coding, and qualitative coding materials were maintained throughout the review process.

To maximize coverage and representativeness, five major electronic databases were searched, yielding an initial total of 3400 records. The databases and initial retrieval counts were as follows: Web of Science Core Collection (WOS, *n* = 883), Scopus (*n* = 2147), the American Psychological Association databases PsycInfo and PsycArticles (*n* = 84), the Education Resources Information Center (ERIC, *n* = 45), and the Répertoire International de Littérature Musicale (RILM, *n* = 241). The search strategy was constructed around three core concepts: “artificial intelligence,” “music learning,” and “target psychological outcomes.” The complete database-specific search strategies are provided in Supplementary Materials, Table S1.

**Figure 1:**
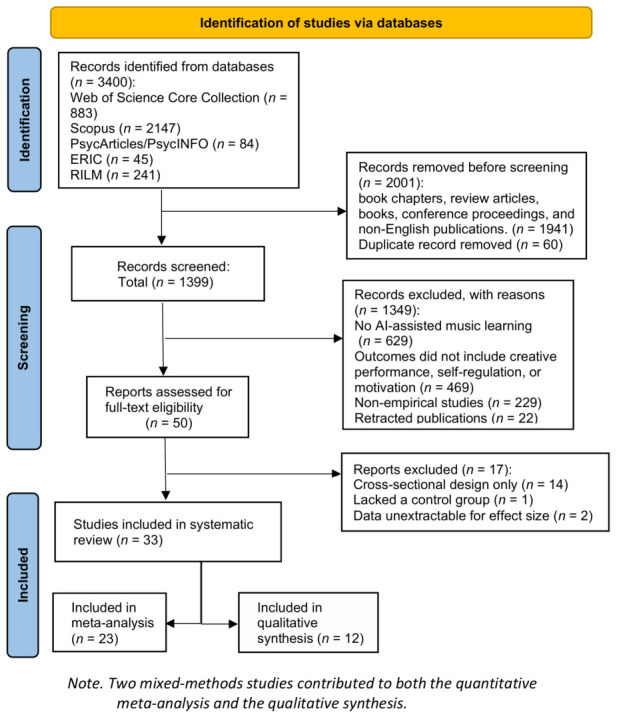
PRISMA diagram.

The screening process consisted of three sequential steps: removal of records before screening, title-and-abstract screening, and full-text eligibility assessment. First, records that were clearly outside the review scope because of publication type or language were removed, and duplicate records were identified through Excel-assisted comparison and manual cross-checking (see Figure 1). Two independent researchers checked titles, authors, publication years, sources, and DOI information to minimize the risk of missing duplicate records. After this step, 1399 records remained for title-and-abstract screening.

Next, the two researchers independently reviewed the titles and abstracts of these records and excluded studies that clearly did not meet the inclusion criteria, including studies without AI-assisted music learning, studies without relevant outcome measures, non-empirical studies, and retracted publications. Finally, the remaining full-text reports were assessed against the predefined inclusion and exclusion criteria, as detailed in Table 1. Any disagreements during screening or eligibility assessment were resolved through discussion; if consensus could not be reached, a third senior researcher was consulted for adjudication.

To accommodate different methodological traditions, the included studies were then allocated to two parallel analytical pathways. Specifically, 23 experimental or quasi-experimental studies with complete statistical data were included in the quantitative meta-analysis (W. Li et al., 2025; Yin & Guo, 2025; L. Zhang, 2025; Xin, 2024; Yuan, 2024; H. Liu & Guo, 2025; Ou et al., 2025; K. Wang & Zhang, 2025; S. Wang, 2025; Gai, 2025; Lv, 2023; B. Liu & Liao, 2025; X. Wang, 2025; P. Liu, 2025; C. Zhang & Li, 2025; X. Liu et al., 2022; Xu & Xu, 2024; N. Li & Wu, 2025; Tao, 2026; Zhuang & Li, 2025; Kuldanov et al., 2025; Baxi et al., 2025; B. Dash et al., 2025), whereas 12 studies containing qualitative designs were included in the qualitative synthesis. It should be noted that two of the final included studies used mixed-methods designs and therefore contributed to both the quantitative and qualitative components of the review.

### 3.2. Scope of the Review and Treatment of Educational Contexts

Music education encompasses highly diverse settings, ranging from early-childhood initiation and formal classrooms to instrumental training and informal learning. The present review does not aim to cover all forms of AI use in music education. Instead, it focuses on empirical studies of AI-assisted music learning that reported learner outcomes related to learning motivation, SRL, or creative performance. Therefore, studies concerned only with AI music-generation technology, music information retrieval, software development, or general discussions of AI in music education without learner-outcome evidence were outside the scope of this study.

Even within this outcome-focused scope, the included studies span diverse contexts (e.g., instrumental practice, composition, and human–AI interactive performance). Due to the limited number of studies per context and the overlapping nature of the interventions, we did not treat these educational settings as separate quantitative subgroups. Instead, this contextual diversity serves as an essential interpretive backdrop. The pooled quantitative estimates should be understood as broad, context-sensitive evidence patterns rather than uniform effects applicable to all music education, while our qualitative synthesis further unpacks how AI support is implemented across these specific settings.

### 3.3. Outcome Extraction Categories

(1) Motivation-related outcomes: This category included a range of proximal subjective indicators reflecting learners’ motivational states, primarily academic motivation, learning interest, self-efficacy, and perceived task value. According to self-determination theory and expectancy–value theory, learners’ motivation in music learning—especially in complex artistic creation or performance tasks—does not operate as a single component, but rather as a constellation of interrelated dimensions, including interest, competence beliefs, value judgments, and engagement states (Evans, 2015; Sin et al., 2022). In AI-assisted contexts, generative and feedback-based tools often reduce barriers to entry, provide immediate feedback, and enhance learners’ sense of control, thereby preferentially activating interest, self-efficacy, and willingness to engage. Accordingly, these theoretically related and empirically interlinked subdimensions were grouped under “motivation-related outcomes” in order to capture, as comprehensively as possible, the awakening effect of AI on learners’ motivational systems at the early stage of learning.
(2) SRL outcomes: This category focused on learners’ self-monitoring and strategic regulation during the learning process, including metacognitive ability, deep learning strategies, information processing and cognitive regulation, as well as critical reflection. Because these variables jointly reflect learners’ active regulation in goal setting, process monitoring, strategy adjustment, and reflective evaluation, they were classified as self-regulation-related outcomes.
(3) Creative performance outcomes: This dimension captures indicators of higher-order divergent thinking and the quality of aesthetic output. Specifically, these outcomes include creative products, expert or teacher ratings, standardized creativity indicators, and creative self-beliefs, in line with the view that creativity involves both originality and effectiveness (Runco & Jaeger, 2012).

### 3.4. Coding Criteria for Core Moderators

Two independent researchers systematically coded key moderator variables, resolving disagreements through discussion or a third reviewer. All results were cross-checked before entry to ensure consistency.

To address sparse data distribution in the quantitative moderation testing, learning outcomes were regrouped into two overarching categories: affective–motivational outcomes and higher-order cognition (combining SRL and creative performance). Conversely, the qualitative synthesis examined SRL and creativity separately to preserve specific mechanistic insights. This regrouping was used only for moderator analyses and was motivated by the limited number of available studies within some outcome-by-moderator cells. Conceptually, SRL and creative performance are not identical. SRL emphasizes goal setting, monitoring, strategic adjustment, and reflection, whereas creative performance emphasizes originality, aesthetic judgment, and the quality of generated products. However, both require learners to move beyond simple task completion and engage in higher-level monitoring, selection, revision, and evaluative judgment. For this reason, they were combined pragmatically as “higher-order cognition” only when testing moderators. To avoid conceptual overgeneralization, the main-effect models estimated SRL and creative performance separately, and the qualitative synthesis also interpreted their implementation pathways and learner experiences separately. Findings for higher-order cognition should therefore be read as pragmatic moderator-level evidence rather than as evidence that SRL and creativity respond to AI support in the same way.

Initially, learners’ prior ability and task cognitive complexity were coded into three levels (low, medium, and high). Low-complexity tasks involved explicit goals and basic imitation; medium tasks required flexible rule application and continuous monitoring; high-complexity tasks were open-ended, demanding the deep integration of multiple musical elements and imposing a high cognitive load.

However, to prevent sparse data cells and improve statistical power—particularly when testing interactions—these variables were collapsed into a binary contrast (low/medium vs. high) for the final moderator analyses. This allowed for a clearer comparison between high-level and non-high-level conditions. Nevertheless, the original three-level coding was retained during data extraction, and the binary contrast should not be interpreted as evidence that low and medium levels are theoretically identical.

Two additional study-level subgroup variables were coded for supplementary analyses. Measurement tool type was coded as standardized/established tools versus self-developed or unclear tools. Studies using established scales, standardized tests, clearly defined performance assessments, or established expert-rating frameworks were classified as standardized/established. Studies using self-developed questionnaires, local rating rubrics, teacher/expert ratings with limited psychometric reporting, system-generated indicators, or tools with unclear validation were classified as self-developed/unclear.

AI support type was coded according to the dominant pedagogical function of the technology: evaluative feedback only, generative support only, adaptive/personalized only, or hybrid AI support. Because some AI-support categories contained fewer than three studies within specific outcome domains, these cells were retained descriptively but were not formally interpreted in the subgroup analysis. The extracted effect-size classification table, including the classification of outcome domains and moderator-related coding information, is provided in Supplementary S2.

### 3.5. Effect-Size Computation and Statistical Analysis Strategy

#### 3.5.1. Effect-Size Computation

Hedges’ *g* was used as the common effect-size metric across all three research questions in order to correct for small-sample bias (Hedges, 1981). Whenever possible, effect sizes were calculated directly from post-intervention means, standard deviations, and sample sizes for the AI and comparison groups. When these statistics were unavailable, equivalent information—including *t* values, *F* values, *p* values, and standard errors—was converted into Hedges’ *g* using standard meta-analytic procedures. For studies reporting pretest–posttest data, the most direct calculation route was used, with priority given to intervention-related change information when clearly reported. All effect sizes were coded in a positive direction, such that positive values always indicated more favorable outcomes or greater improvement under the AI-assisted condition. The extracted effect sizes and outcome classifications used for the meta-analytic models are provided in Supplementary S3.

#### 3.5.2. Model Specification for Main and Moderating Effects

Because many primary studies reported multiple eligible outcome indicators from the same sample, the present study used three-level random-effects models to appropriately account for the statistical dependence among effect sizes (Assink & Wibbelink, 2016; Cheung, 2014; Van den Noortgate et al., 2013). In these models, Level 1 represented sampling variance, Level 2 represented within-study heterogeneity, and Level 3 represented between-study heterogeneity. All models were estimated using restricted maximum likelihood (REML). Model specifications for each research question were as follows.

(1) For RQ1, separate intercept-only models were fitted for creative performance, SRL, and learning motivation in order to preserve the theoretical independence of the three outcome domains and estimate the average intervention effect for each domain. At the same time, to improve the transparency of cross-domain comparisons and to avoid giving disproportionate weight to studies reporting multiple similar indicators, a descriptive synthesis was also conducted by first averaging effect sizes within studies and then summarizing the overall weighted means, medians, and related descriptive statistics.
(2) For RQ2, separate three-level mixed-effects meta-regression models were fitted for motivation and higher-order cognition (defined as the combination of creativity and SRL) in order to test the main effects of task cognitive complexity and prior ability, as well as their joint moderating effect through the interaction term.
(3) For RQ3, a three-level meta-regression model was specified, including intervention dose, outcome domain, and their interaction, in order to examine whether dose-related patterns differed across outcome types (motivation and higher-order cognition). To facilitate the interpretation of potential threshold-like patterns, predicted marginal effects were further estimated across the three dose levels.

Across these models, moderator analyses were consistently reported using regression coefficients (*b* or interaction coefficients), standard errors, 95% confidence intervals, and two-sided *p* values. Residual heterogeneity was described using the Level 2 and Level 3 variance components together with multilevel versions of *Q* and *I*^2^ when informative (Cheung, 2014; Higgins et al., 2003).

Supplementary subgroup analyses were further conducted for measurement tool type and AI support type. Given the small number of studies in several subgroup cells, only cells with sufficient study representation were formally interpreted, and all subgroup findings were treated as exploratory.

#### 3.5.3. Robustness Checks, Sensitivity Analyses, and Publication Bias

To comprehensively evaluate the stability of the findings, the following procedures were conducted. Publication bias diagnostics were not treated as a central inferential procedure in the present study. This is because several analyses were based on multilevel structures, dependent effect sizes, or relatively small numbers of independent study clusters within specific outcome domains or moderator cells, conditions under which funnel-plot asymmetry and Egger-type tests are of limited interpretive value. Instead, the stability of the findings was primarily evaluated through a series of robustness and sensitivity analyses, including cluster-robust estimation, leave-one-study-out analyses, and the exclusion or adjustment of extreme and nonstandard effect sizes.

(1) Supplementary RVE analyses. To determine whether the treatment of dependent effect sizes affected the moderator findings, both RQ2 and RQ3 were additionally re-estimated using inverse-variance-weighted meta-regression with study-clustered robust standard errors, following the literature on robust variance estimation (RVE). These analyses were treated as supplementary robustness checks to the primary three-level models (Hedges et al., 2010; Pustejovsky & Tipton, 2022; Tipton, 2015; Tipton & Pustejovsky, 2015).
(2) Sensitivity analyses. These included leave-one-study-out analyses; targeted exclusion of effect sizes flagged during extraction as rough estimates or nonstandard approximations; deletion, winsorization, or truncation of extreme effect sizes (e.g., absolute values greater than 3.00); and direction-recoding checks for negatively oriented subscales.
(3) Publication bias. The present review included multiple effect sizes from the same studies and used multilevel models to account for their dependence. Therefore, publication-bias diagnostics were conducted at the outcome-domain level rather than by pooling all effect sizes together. Funnel plots were used to visually inspect small-study effects, Egger’s regression was used to provide a statistical test of funnel-plot asymmetry, and trim-and-fill analysis was used as a supplementary adjustment-based diagnostic (Duval & Tweedie, 2000; Egger et al., 1997). Because some outcome domains included a limited number of independent study clusters, these diagnostics were interpreted as exploratory evidence rather than as definitive tests of publication bias.

#### 3.5.4. Software and Decision Criteria

All statistical analyses were conducted in the R environment, and the three-level models were estimated using the metafor package (Viechtbauer, 2010). Statistical significance was evaluated at α = 0.05 using two-tailed tests. In interpreting the findings, the study considered not only *p*-values, but also the absolute magnitude, precision, and consistency of effects across outcome domains and robustness models. The core R scripts used for the main meta-analytic analyses of the research questions are provided in Supplementary S4.

### 3.6. Qualitative Synthesis Analysis

To address RQ4, the qualitative synthesis adopted a structured thematic approach, focusing on how AI support was implemented in music learning and how learners experienced such support.

First, two independent researchers extracted process- and experience-related information from the included qualitative and mixed-methods studies. The extracted information included the author(s), title, research design, specific music learning activity and AI type, target outcome domain (motivation, SRL, or creativity), form of AI assistance, concrete implementation strategy, learner experience, and original textual evidence.

Second, the coding process was conducted in three stages. In the first stage, “implementation codes” were assigned to identify the pedagogical role of AI in music learning, such as evaluative feedback, generative support, or adaptive personalization. In the second stage, “experience codes” were assigned to capture learners’ responses during AI-supported learning, such as increased interest, reduced anxiety, self-monitoring, critical filtering, or creative exploration. In the third stage, the initial codes were compared across studies and grouped into broader themes, resulting in three main implementation pathways.

Third, because the qualitative synthesis in this study was used primarily to complement the meta-analytic findings by providing contextual and mechanism-oriented explanations of AI implementation and learner experience, rather than as a standalone qualitative systematic review, no formal score-based quality appraisal using a single appraisal tool was conducted. The qualitative evidence contributed by the included studies was heterogeneous in form, including semi-structured interviews, open-ended responses, reflective logs, classroom observations, interaction records, and brief descriptions of learner experience. To avoid applying an inappropriate uniform scoring system to different types of evidence, the interpretive contribution of each qualitative source was considered according to three aspects: the clarity of the qualitative data source, the transparency of the analytic procedure, and the directness of evidence concerning AI implementation or learner experience.

Overall, studies that clearly reported interviews, open-ended responses, reflective logs, or systematic observations and provided direct evidence of learner experience were given greater interpretive weight in theme development. Studies that relied mainly on author interpretation, classroom description, or limited experiential statements were used more cautiously and treated primarily as contextual support. This approach allowed the synthesis to retain useful evidence concerning AI implementation and learner experience while avoiding overinterpretation of weaker qualitative materials.

Finally, any disagreements during coding and theme generation were resolved through discussion; when consensus could not be reached, a third reviewer was consulted for adjudication.

## 4. Results

### 4.1. Risk of Bias Assessment and Conflict-of-Interest Statements

**Figure 2:**
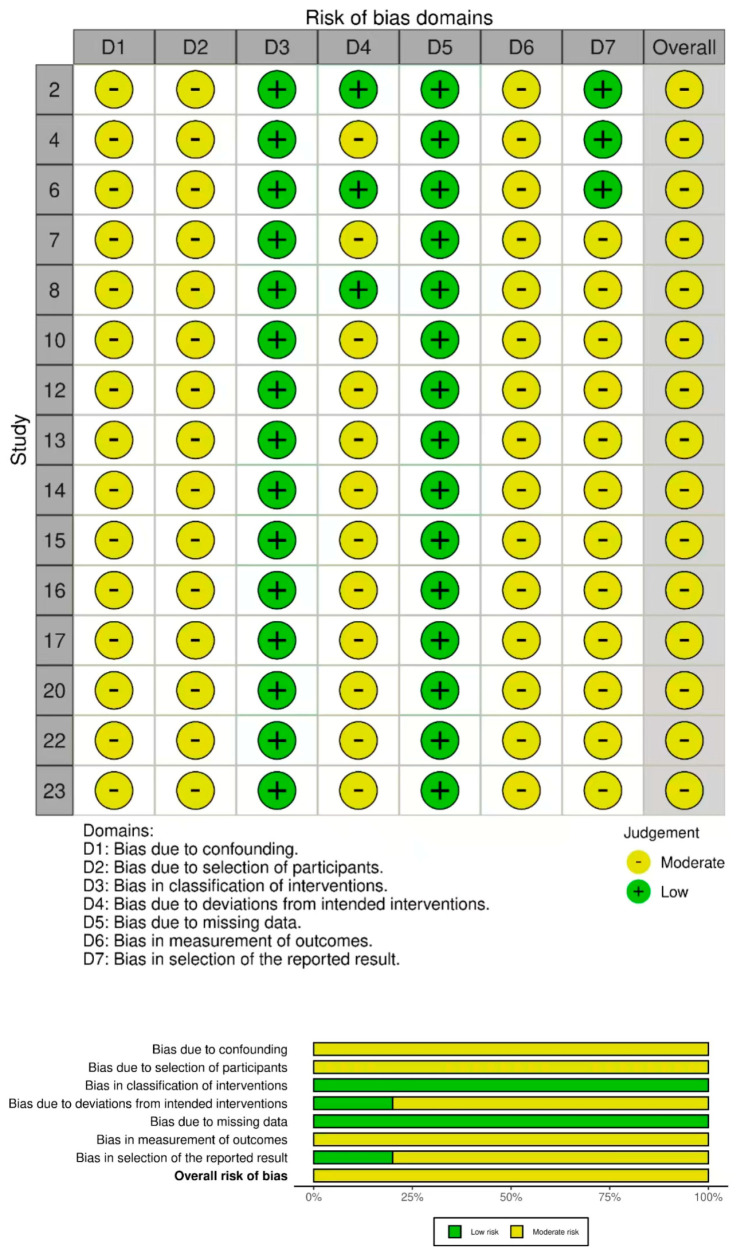
Risk-of-bias assessment of the included CCT studies.

**Figure 3:**
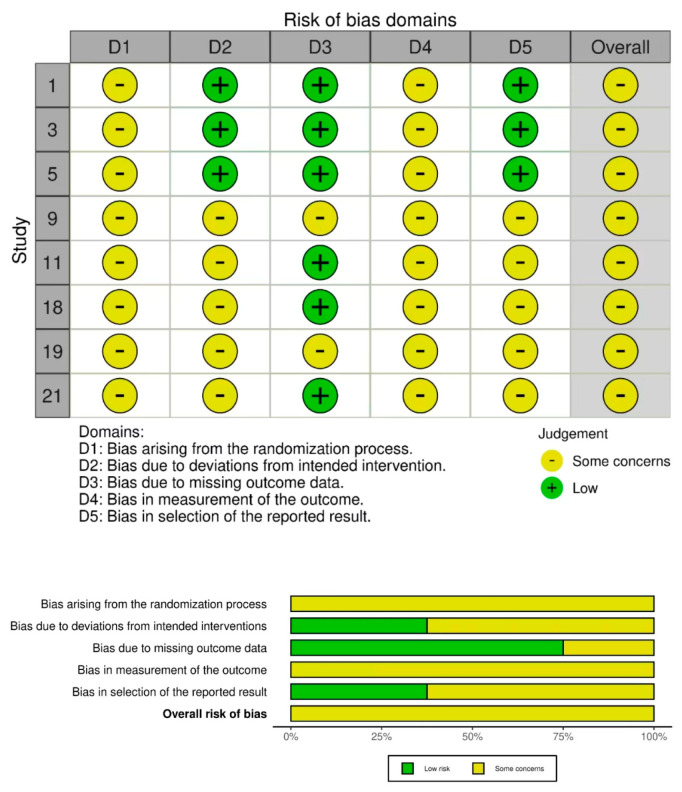
Risk-of-bias assessment of the included RCT studies.

Risk-of-bias assessment was conducted for all studies included in the meta-analysis (detailed characteristics of these 23 included studies are presented in Table S2 of Supplementary Materials). Specifically, the 15 controlled clinical trials (CCTs) were evaluated using the ROBINS-I framework (see Figure 2), whereas the 8 randomized controlled trials (RCTs) were assessed using the RoB 2 tool (see Figure 3). Overall, the methodological quality of the included studies was at a moderate level. Although no study was rated as high risk, there was also a lack of high-quality studies that could be considered low risk overall. Across bias domains, measurement bias, insufficient implementation of randomization, and inadequate control of confounding factors emerged as the most common methodological limitations. Accordingly, the findings should still be interpreted with caution.

In addition, with regard to independence screening, the 23 empirical studies included in this review generally demonstrated a relatively high degree of objectivity. The systematic review did not identify any major potential conflicts of interest involving specific software developers or commercial profit-oriented institutions. With respect to funding disclosure, the vast majority of studies either explicitly stated that they had not received any specific financial support or did not report funding information.

### 4.2. Overall Intervention Effects of AI-Assisted Music Learning

**Figure 4:**
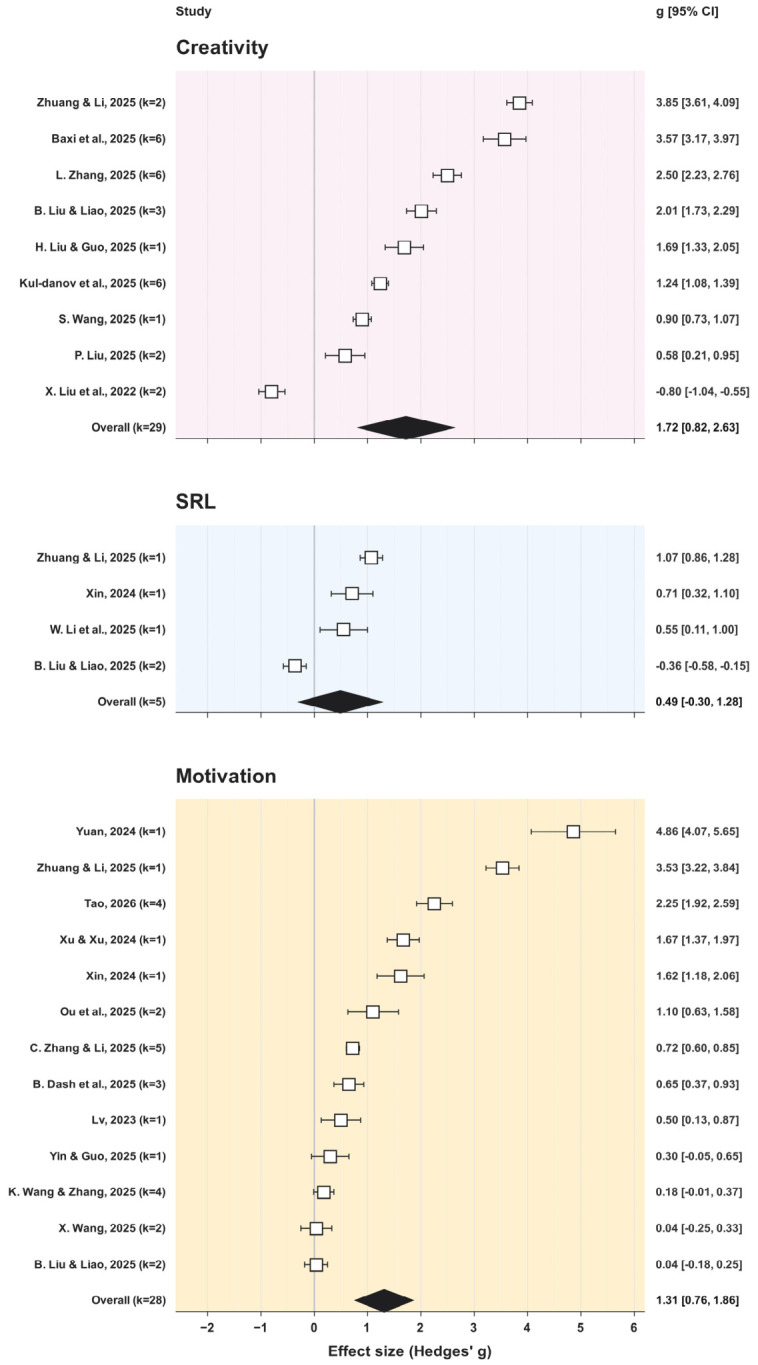
Three-Level Meta-Analysis Forest Plot of Main Effects (Zhuang & Li, 2025; Baxi et al., 2025; L. Zhang, 2025; B. Liu & Liao, 2025; H. Liu & Guo, 2025; Kuldanov et al., 2025; S. Wang, 2025; X. Wang, 2025; P. Liu, 2025; X. Liu et al., 2022; Xin, 2024; W. Li et al., 2025; Yuan, 2024; Tao, 2026; Xu & Xu, 2024; Ou et al., 2025; C. Zhang & Li, 2025; B. Dash et al., 2025; Lv, 2023; Yin & Guo, 2025; K. Wang & Zhang, 2025).

Based on the current body of included studies, AI-assisted music learning showed positive trends across creativity, learning motivation, and self-regulated learning (SRL), although the magnitude and robustness of these effects differed across outcome domains. At the descriptive level, creativity showed the largest average effect, followed by learning motivation, whereas the overall effect for SRL was comparatively smaller. Specifically, creativity was represented by 9 studies (*N* = 1790), with a study-level aggregated weighted mean effect size of *g* = 1.781, a simple mean of *g* = 1.726, and a median of *g* = 1.690 (see Figure 4). Learning motivation was represented by 13 studies (*N* = 1923), with a weighted mean effect size of *g* = 1.556, a simple mean of *g* = 1.344, and a median of *g* = 0.724. SRL was represented by 4 studies (*N* = 812), with a weighted mean effect size of *g* = 0.583, a simple mean of *g* = 0.492, and a median of *g* = 0.632.

The formal random-effects meta-analysis further indicated that the three domains did not show the same degree of statistical support. A significant positive overall effect was found for creativity (*g* = 1.206, 95% CI [0.056, 2.355], 95% PI [−2.607, 5.019], *p* = .042), and a similarly significant positive effect was found for learning motivation (*g* = 1.278, 95% CI [0.412, 2.144], 95% PI [−1.563, 4.119], *p* = .007). In contrast, the pooled effect for SRL was positive but did not reach statistical significance in the main model (*g* = 0.565, 95% CI [−0.207, 1.338], 95% PI [−2.030, 3.160], *p* = .102). Thus, the main model provided clearer statistical support for creativity and motivation than for SRL. Because all three prediction intervals were wide and crossed zero, these pooled effects should be interpreted as broad average tendencies across heterogeneous contexts rather than as stable effects expected across all AI-assisted music learning settings.

At the same time, the robustness of these findings differed across domains. Learning motivation not only remained significant in the main model, but also showed directionally consistent and statistically significant results across the cluster-robust model and multiple sensitivity analyses, indicating that it was the most robust outcome domain. By contrast, although creativity was significant in both the main model and the cluster-robust model, its heterogeneity was extremely high, and the leave-one-study-out analysis showed that the result became non-significant after removing Zhuang and Li (2025), suggesting that the average creativity effect was more sensitive to individual studies and model specifications. For SRL, the effect remained non-significant in both the main model and the cluster-robust model, and its conclusion changed under alternative coding or study-removal scenarios.

Supplementary subgroup analyses were conducted to explore possible sources of heterogeneity. For learning motivation, positive estimates were found across measurement types, including standardized/established tools (*g* = 1.356, 95% CI [0.304, 2.407], *k* = 8) and self-developed or unclear tools (*g* = 1.221, 95% CI [−0.024, 2.466], *k* = 5). Motivation effects were also positive for evaluative-feedback AI (*g* = 1.137, 95% CI [0.252, 2.023], *k* = 4) and generative-support AI (*g* = 1.815, 95% CI [−0.094, 3.723], *k* = 5). For creative performance, larger estimates were observed for self-developed or unclear tools (*g* = 1.456, 95% CI [0.289, 2.623], *k* = 6) and generative-support AI (*g* = 2.109, 95% CI [0.363, 3.855], *k* = 3), whereas hybrid AI support showed a smaller and less precise estimate (*g* = 0.719, 95% CI [−0.318, 1.756], *k* = 4). SRL subgroup cells contained fewer than three studies and were not formally interpreted. These subgroup findings should be interpreted as exploratory rather than confirmatory.

These average effects should be interpreted in relation to the diverse music learning contexts represented in the included studies. Motivation-related effects were often observed in settings where AI lowered the threshold for participation or made practice more immediately responsive, such as intelligent piano learning, AR-supported piano practice, violin practice applications, and AI-enhanced early-childhood music activities. Creativity-related effects were more often linked to composition, songwriting, generative music production, and human–AI co-creation tasks. In contrast, SRL-related outcomes appeared to depend more on whether AI feedback was repeatedly used for goal setting, monitoring, strategy adjustment, and reflection. Thus, the pooled effects should be read as context-sensitive average patterns rather than as uniform effects across all forms of music education.

### 4.3. Interactive Moderating Effects of Task Complexity and Prior Ability

To address RQ2, we examined whether task cognitive complexity (low/medium vs. high) and learners’ prior ability (low/medium vs. high) jointly moderated the effects of AI-supported music learning on motivation and higher-order cognition. Higher-order cognition was operationalized as self-regulated learning and creativity. Given the non-independence of multiple effect sizes extracted from the same study, we treated the three-level meta-regression as the primary analytic model and used a cluster-robust weighted meta-regression as a supplementary robustness check. Overall, evidence for a task complexity × prior ability interaction was limited and outcome-specific: tentative support emerged for motivation, whereas no robust interaction was observed for higher-order cognition.

**Figure 5:**
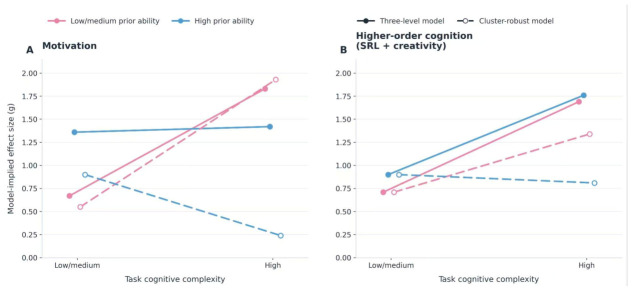
Predicted effects for the interaction between task cognitive complexity and prior ability. (**A**) Motivation; (**B**) Higher-order cognition (SRL + creativity). Solid lines indicate the primary three-level model, and dashed lines indicate the supplementary cluster-robust model.

For motivation, the interaction between task cognitive complexity and prior ability was negative but not statistically significant in the primary three-level meta-regression, *b* = −1.10, *SE* = 1.65, *z* = −0.67, *p* = .506, 95% CI [−4.34, 2.14] (see Table 1 and Figure 5). Residual heterogeneity remained substantial, with level-2 variance *τ*^2^{L2} = 0.74 and level-3 variance *τ*^2^{L3} = 1.69, corresponding to total *I*^2^ = 97.72% (level 2 = 29.70%; level 3 = 68.02%). The residual heterogeneity test was significant, *Q*E(24) = 784.56, *p* < .001. In the supplementary cluster-robust model, however, the interaction for motivation reached statistical significance and remained in the same negative direction, *b* = −2.03, *SE* = 0.89, *z* = −2.29, *p* = .022, 95% CI [−3.77, −0.29]. Descriptively, this pattern suggests that high-complexity tasks may be more favorably associated with motivational outcomes among learners with lower prior ability than among those with higher prior ability.

For higher-order cognition, the interaction term was likewise negative and non-significant in the primary three-level model, *b* = −0.12, *SE* = 3.11, *z* = −0.04, *p* = .969, 95% CI [−6.21, 5.97]. Again, residual heterogeneity was substantial, with *τ*^2^_{L2} = 2.81 and *τ*^2^_{L3} = 1.38, corresponding to total *I*^2^ = 97.91% (level 2 = 65.66%; level 3 = 32.25%). The residual heterogeneity test was significant, *Q*E(29) = 2025.77, *p* < .001. The supplementary cluster-robust model produced the same directional pattern but also failed to detect a statistically significant interaction, *b* = −0.72, *SE* = 0.83, *z* = −0.87, *p* = .386, 95% CI [−2.34, 0.91]. Thus, for higher-order cognition, the available evidence does not support a reliable interaction between task complexity and prior ability.

Educationally, these findings suggest that learner–task fit may be especially relevant when AI is used in open-ended or cognitively demanding music tasks, such as composition, arrangement, improvisation, or human–AI co-creation. However, because the interaction was significant only for motivation in the supplementary robust model, and not in the primary three-level model or for higher-order cognition, this finding should be interpreted as a hypothesis-generating contextual implication rather than as robust evidence for a general learner–task fit principle.

### 4.4. Dose-Related Patterns of AI-Assisted Music Learning

For the dose-based analyses, intervention duration was classified into short-term (≤7 weeks), medium-term (8–12 weeks or one academic semester), and long-term (≥13 weeks). Based on the dataset (22 studies, 87 effect sizes, *N* = 3318), dependence-robust meta-regression revealed that the moderating effect of intervention dose varied significantly by outcome domain.

**Figure 6:**
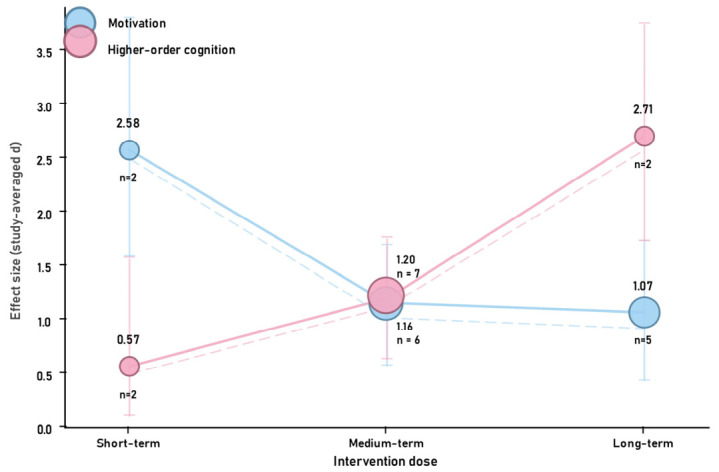
Dose effects by outcome type.

Specifically, dose significantly moderated higher-order cognitive outcomes, highlighted by a significant long-term × higher-order cognition interaction (*b* = 2.24, *p* < .001) (see Table 2 and Figure 6). However, this coefficient should be interpreted cautiously because the long-term higher-order cognition subgroup contained only a small number of studies and included several very large effect sizes. The dose slope was significant for higher-order cognition (*b* = 1.20, *p* < .001) but not for motivation (*b* = −0.07, *p* = .888). Predicted marginal effects confirmed this divergence: motivational outcomes plateaued or slightly declined across short-, medium-, and long-term interventions (1.03, 0.82, and 0.79, respectively), whereas higher-order cognitive outcomes increased substantially across the same levels (0.56, 0.69, and 2.23).

Study-averaged descriptive statistics mirrored this pattern. Motivational outcomes peaked in short-term interventions (*g* = 2.58, 2 studies) and stabilized in medium- (*g* = 1.16, 6 studies) and long-term interventions (*g* = 1.07, 5 studies). Conversely, higher-order cognitive outcomes progressively increased from short- (*g* = 0.57, 2 studies) to medium- (*g* = 1.20, 7 studies) and long-term interventions (*g* = 2.71, 2 studies). This descriptive pattern was consistent with the model-based estimates, suggesting that motivational outcomes may be more immediately responsive to short-term AI exposure, whereas higher-order cognitive outcomes may become more visible under longer interventions.

Given the presence of unusually large effects (13 effect sizes > |*d*| = 3.00), multiple sensitivity analyses were conducted. Winsorizing extreme values at *d* = 3.00 yielded a robust pooled effect (*g* = 1.18, 95% CI [0.78, 1.57]) and higher-order dose slope (*b* = 1.10, *p* < .001). Completely removing these extreme values (*g* = 0.92; slope *b* = 0.93, *p* = .0007) or excluding rough/non-standard extractions (*g* = 1.14 to 1.16; slope *b* = 0.95, *p* = .0485) consistently upheld the substantive conclusions. Finally, leave-one-study-out analyses confirmed that the pooled effect (*g* range: 1.20–1.41) and the long-term advantage for higher-order cognition remained statistically significant across all iterations. Thus, the dose-related pattern for higher-order cognition was not solely driven by a single study or by extreme values.

Educationally, this pattern may correspond to different forms of AI integration. Short-term AI activities, such as introductory workshops, trial lessons, or brief creative tasks, may be sufficient to generate novelty, interest, and perceived accessibility. By contrast, longer interventions, such as semester-based composition projects, sustained instrumental practice with AI feedback, or repeated human–AI creative cycles, may provide more opportunities for learners to internalize strategies, revise outputs, and engage in reflective regulation. Nevertheless, because the long-term higher-order cognition subgroup contained relatively few studies and several large effects, this finding should be interpreted as provisional trend-based evidence rather than as evidence of a stable dose–response relationship.

### 4.5. Implementation, Experience, and Patterns of AI Support

The qualitative synthesis included 12 studies that provided process- or experience-related evidence on AI-supported music learning (Addessi & Pachet, 2005; Baxi et al., 2025; Cui, 2023; Dong et al., 2024; W. Li, 2022; Lin et al., 2025; Nakajima et al., 2026; Ou et al., 2025; Roldan-Cardona et al., 2025; Sun, 2026; Wani et al., 2025; Yao, 2025). This section uses the qualitative synthesis to provide a contextual interpretation of the preceding quantitative findings. Specifically, the quantitative analyses identified the overall effects and boundary conditions of AI-assisted music learning across motivation, SRL, and creative performance, whereas the qualitative synthesis further clarifies how these statistical patterns may be implemented and experienced in concrete music learning processes. By examining the pedagogical roles of AI support, learners’ responses, and task contexts, this section helps explain why motivational outcomes appeared relatively robust, why SRL outcomes remained less stable, and why creativity-related effects showed substantial heterogeneity.

Although SRL and creative performance were combined under higher-order cognition in the quantitative moderator analyses, they were examined separately in the qualitative synthesis because their implementation pathways and learner experiences were not identical (Detailed characteristics of the 12 included studies are presented in Table S3 of Supplementary Materials).

#### 4.5.1. Pathways of AI Support Implementation

First, evaluative-feedback AI provided immediate support by constructing a “performance–monitoring–feedback” loop. For example, a web-based Musical Instrument Digital Interface (MIDI) platform used gesture tracking and Follow mode for real-time comparative instruction (S2), an intelligent piano system used a convolutional neural network to automatically slow down and provide prompts when children made mistakes (S5), augmented reality applications superimposed virtual expert movements onto the keyboard to provide audiovisual guidance (S6), and Violy quantified violin practice performance through objective scoring (S9).

Second, generative-support AI expanded expressive possibilities through human–AI co-creation. In a STEAM project, students used block-based programming to control a social robot’s movements, voice, and sound generation, and completed situated creation in a human–AI collaborative musical theater context (S1). In composition and interdisciplinary teaching contexts, learners used AIVA, Suno, or Transformer-XL to rapidly generate melodic, harmonic, lyrical, or sound-effect materials based on style, emotion, or lyric themes (S3, S4, S7, S10). The Continuator, in turn, enabled improvised turn-taking through “musical mirroring” (S12).

Third, adaptive and personalized AI support was manifested more clearly in its alignment with individual learning needs. On the one hand, learners actively used AI as a resource for problem solving and learning support (S8). On the other hand, in early childhood education, AI also created more adaptive learning environments through interactive games such as Genially (S11).

#### 4.5.2. Learners’ Subjective Experiences in Human–AI Collaborative Contexts

At the level of subjective experience, most studies suggested that learners often moved from initial novelty activation toward a stronger sense of competence, control, or sustained willingness to participate. At the motivational level, learners often began with feelings of surprise, excitement, and enjoyment (S1, S7, S12), which gradually developed into more stable participation intentions through continued interaction. Gamified feedback reduced resistance to practice (S5), while the shift from listener to creator significantly enhanced classroom engagement (S4).

At the level of self-regulation, AI provided learners with a sense of security and control, enabling them to engage in independent error correction and strategy adjustment even in the absence of the teacher (S6, S9). Through information verification, proactive questioning, and task monitoring, learners also demonstrated stronger regulatory capacity (S8). In more complex creative tasks, this experience further took the form of repeated psychological negotiation between algorithmic suggestions and personal intuition (S3, S10).

At the level of creative performance, learners reported a noticeable reduction in blank-page anxiety (S10). Because AI provided multiple versions of melodies, harmonies, and stylistic alternatives, learners also experienced greater creative freedom, more room for trial and error, and stronger enjoyment in experimentation (S3, S4, S7).

#### 4.5.3. Common Patterns: The Underlying Logic of AI Support

Taken together, the 12 studies revealed three relatively consistent underlying patterns.

The first was the lowering of barriers and the enhancement of willingness to participate. Through automatic error correction (S5, S9), gesture mapping (S6), multimodal prompting (S2), or the rapid generation of melodies and lyrics (S4, S7), AI reduced the entry cost of music learning and creation, thereby shifting learners from hesitation toward active engagement (S2, S11).

The second was novelty activation followed by reflective filtering. AI outputs often first stimulated inspiration and attention through their novelty (S7, S10, S12), and then prompted learners to enter a reflective phase involving critical listening, version selection, and iterative revision (S3, S7, S10), thereby reshaping the cognitive pathway of learning.

The third was human–AI collaboration rather than technological substitution. This was evident both in teachers’ guidance within AI-supported classrooms (S2, S4) and in students’ rejection, modification, and regeneration of AI suggestions (S10).

#### 4.5.4. Key Differences

Clear contextual differences nevertheless emerged across studies. The first concerned differences in cognitive maturity. For young children, AI functioned more like an interactive playmate or responsive partner, mainly sustaining joint attention and eliciting early vocal, rhythmic, and musical responses (S11, S12). For university students and adult learners, by contrast, AI functioned more as an efficiency-enhancing tool and reflective medium, emphasizing academic control, self-efficacy, and ethical boundary awareness (S8, S10).

The second concerned differences in task goals. In practice-oriented tasks, AI was closer to a “coach” or “mirror,” focusing on error correction, monitoring, and efficiency enhancement; learners’ experiences were primarily characterized by a sense of certainty in independent practice (S2, S5, S6, S9). In creative tasks, AI functioned more as a “partner” or “catalyst,” emphasizing inspiration, generation, and comparison; learners’ experiences centered more on aesthetic decision making, overcoming anxiety, and stylistic experimentation (S3, S4, S7, S10). In performance or interactive tasks, AI resembled an “actor” or “collaborative other,” emphasizing responsiveness, co-presence, and situated participation; learners’ experiences, therefore, were more strongly involved in joint attention, social surprise, and immediate engagement (S1, S12).

The third concerned differences in outcomes supported by different AI types. Evaluative-feedback AI mainly focused on self-regulation, efficacy, and practice quality (S2, S5, S6, S9). Through immediate correction and score-based quantification of performance, it helped learners develop a sense of control and improve performance precision during independent practice. Generative-support AI, by contrast, primarily supported creative performance, creative expansion, and motivational activation (S3, S4, S7, S10). By rapidly providing inspirational materials, it reduced creative anxiety and enabled learners to extend their creative boundaries through aesthetic decision-making and critical filtering. Composite/adaptive AI forms (e.g., S1, S11, S12) showed stronger inclusiveness and persistence. Their core advantage lies in dynamically adjusting environmental difficulty in response to the immediate reactions of younger or otherwise specialized learners (adaptive property), while simultaneously assisting melodic generation (generative property) and significantly enhancing vocal participation, motivational stability, and joint attention.

## 5. Discussion

### 5.1. Main Effects

The present study found that AI-assisted music learning showed an overall positive trend across three outcome domains—creative performance, learning motivation, and SRL—but the degree of statistical support was not equivalent across domains. Specifically, creative performance showed the largest average effect and reached statistical significance in the main model, although this result was accompanied by extremely high heterogeneity and was sensitive to individual studies. By contrast, learning motivation not only reached statistical significance in the main model but also showed the most robust and directionally consistent pattern across alternative model specifications and sensitivity analyses, making it the most stable outcome domain in the present review. SRL, although positive in direction, did not reach statistical significance in the pooled analysis, and its conclusion varied depending on coding strategies and sensitivity conditions, indicating that the current evidence base for SRL remains relatively preliminary and should be interpreted with caution. At the same time, the very high heterogeneity observed across several models means that these pooled effects should not be treated as stable or context-invariant estimates. The wide prediction intervals further support this cautious interpretation, indicating that future studies may produce effects ranging from negligible or negative to very large positive effects. Rather, they summarize average tendencies across studies that differed in educational level, music learning task, AI tool, intervention duration, comparison condition, and measurement approach. This is particularly important for creative performance, where the average effect was large but more sensitive to individual studies and study composition. The supplementary subgroup analyses further suggest that part of the heterogeneity may be related to measurement choices and the dominant pedagogical role of AI support, although these patterns remain exploratory because several subgroup cells contained few studies.

This differentiated pattern aligns closely with specific music learning contexts. In practice-oriented settings (e.g., instrumental or MIDI learning), AI primarily functions as an evaluative tool. Its immediate, visual, and gamified feedback may help strengthen learners’ interest, perceived control, and willingness to continue practicing, which helps explain why motivation showed relatively stable evidence. However, this feedback loop does not automatically translate into SRL, which requires sustained cycles of goal-setting and strategy adjustment, explaining the positive yet less stable SRL outcomes.

Conversely, in creative contexts (e.g., composition and human–AI co-creation), AI acts as a generative resource rather than a corrective tool. By providing musical materials for learners to revise and recombine, AI may expand the exploratory idea space and lower entry barriers to creative production, which helps explain the positive but highly heterogeneous creativity effects. Ultimately, these findings highlight context-specific impacts across feedback-driven practice, creative production, and self-regulation, rather than a uniform effect of AI on music education as a whole.

This overall pattern is broadly consistent with the existing AI-in-education literature. Previous studies have shown that AI support often produces a pattern of overall positive but differentiated effects, rather than yielding uniform gains across all outcome domains. Meta-analytic evidence has shown that generative artificial intelligence (GenAI) can significantly enhance students’ motivation and engagement (Xia et al., 2025). By contrast, the effects of AI on SRL appear to be more uneven, with clear variation across metacognitive regulation, motivational/affective regulation, and behavioral regulation (Achuthan, 2025). Similarly, a meta-analysis of experimental studies found that ChatGPT can enhance academic performance, affective–motivational states, and higher-order thinking, but its effects are not consistent across all outcomes (Deng et al., 2025). Against this background, the pattern observed in the present study—namely, relatively robust evidence for learning motivation, comparatively strong but less stable effects for creative performance, and only preliminary evidence for SRL—should not be regarded as inconsistent with prior research. On the contrary, this differentiation is broadly consistent with the judgment advanced in the present theoretical framework, namely that AI may exert a dual-pathway enabling effect, but that different outcomes do not necessarily emerge synchronously.

Accordingly, although AI may create favorable conditions for the development of SRL, such conditions do not necessarily translate into stable, statistically significant, and directly measurable improvements in SRL over the short term. This further suggests that the facilitating effects of AI are unlikely to unfold synchronously across all outcome domains, but are more likely to be jointly moderated by such boundary conditions as learner characteristics, task type, and intervention duration.

### 5.2. Learner–Task Fit

The findings concerning the interaction between prior ability and task cognitive complexity suggest that inferential support for this interaction was generally limited. For motivational outcomes, the interaction between prior ability and task cognitive complexity did not reach significance in the three-level model, although it became significant in the cluster-robust model, indicating some evidence of such an interaction but limited stability. By contrast, for higher-order cognition, the interaction term failed to reach significance in both models, suggesting that the current evidence remains insufficient to establish a stable prior ability–task interaction pattern in this domain. It must be acknowledged that this lack of statistical significance may indicate either a genuinely weak interaction effect or, to a large extent, reflect the limited number of studies in specific cells and the extremely high residual heterogeneity. In some cells, the number of studies (*k*) was particularly small; notably, in the higher-order cognition domain, the two low-complexity combinations each contained only one study. Accordingly, the current findings are better interpreted as directional evidence of adaptation rather than as a rigorously established and unified interaction rule.

At the descriptive level, however, the observed pattern remained broadly consistent with the theoretical framework of the present study, namely that the effectiveness of AI scaffolding is unlikely to be determined by prior ability or task cognitive complexity alone, but rather by the degree of fit between them (Kalyuga et al., 2003; Sweller, 1988; van Merriënboer & Sweller, 2005).

For motivation, all prior ability–task combinations showed positive effects, but the largest descriptive effect emerged for the “high task cognitive complexity × low/medium prior ability” condition rather than the more intuitively expected “low/medium task cognitive complexity × low/medium prior ability” condition. This suggests that, once AI scaffolding is introduced, high-complexity tasks do not necessarily undermine learners’ engagement and may, in some cases, even generate stronger benefits. One possible explanation is that when AI provides generative support, immediate feedback, and personalized path matching in high-complexity tasks, complexity may no longer be experienced purely as greater cost, but rather as a “manageable challenge” (Evans et al., 2024; Feldon et al., 2019). For example, Yuan (2024) found that DeepBach significantly enhanced academic motivation in polyphonic composition, explicitly attributing this to the simplification of a highly complex creative process. In this sense, “fit” at the motivational level may be reflected less in the level of task complexity itself and more in whether AI helps learners reduce threat and enhance perceived control.

By contrast, the pattern for higher-order cognition was more closely aligned with the deep-processing logic emphasized in the theoretical framework. In both the three-level model and the descriptive results, larger higher-order cognitive effects were concentrated primarily in high-complexity tasks, especially in the “high task cognitive complexity × high prior ability” condition. This directional pattern is consistent with the CLT view that cognitive resources should be reallocated toward schema construction and complex processing, and it is also compatible with the basic logic of the expertise reversal effect (Kalyuga et al., 2003; Sweller, 1988; van Merriënboer & Sweller, 2005). At the same time, however, “high task cognitive complexity × low/medium prior ability” also showed relatively large positive effects for higher-order cognition, suggesting that such benefits are not strictly confined to a single “high-ability–high-complexity” pathway. In some studies, AI did not directly replace learners’ task completion, but instead functioned as an external organizer, feedback trigger, and reflective scaffold. Under such conditions, learners with low or medium prior ability may also be able to engage in deeper processing that would otherwise require more mature internal schemas. For example, W. Li et al. (2025) found that AI-supported audio comparison feedback combined with reflection logs and generative dialogic support significantly enhanced metacognitive ability in vocal training, rather than merely improving basic performance skills. In other words, the positive outcomes observed for learners with lower prior ability in high-complexity tasks do not necessarily imply that they possessed internal schemas equivalent to those of higher-ability learners; rather, they may indicate that AI transformed complex processing demands that would otherwise have to be completed independently into externally available scaffolds. This does not contradict the expertise reversal effect; instead, it reinforces the idea that the effectiveness of scaffolding depends on its fit with learners’ schema development (Kalyuga et al., 2003).

In addition, the distribution of studies suggests that motivation-related outcomes appeared more often in low-complexity tasks, whereas higher-order cognition outcomes were more often embedded in high-complexity tasks. To some extent, this pattern aligns with the common design logic of complex learning research: lower-threshold, more clearly structured tasks are more suitable for observing proximal responses such as engagement, interest, and self-efficacy, whereas more open-ended and integrative tasks are better able to capture strategy use, schema construction, and deeper processing (van Merriënboer & Sweller, 2005). However, this natural clustering also resulted in very small sample sizes in some cross-cells (e.g., low complexity × higher-order cognition), which further constrained the possibility of making stronger inferential claims about specific combinations.

Overall, the present evidence does not justify a strong claim that learner–task fit is a stable moderator across all AI-assisted music learning contexts. A more cautious interpretation is that AI scaffolding may be most useful when it helps learners handle complex music tasks without removing their own control over musical decisions. In practical settings, this may occur when AI simplifies a demanding composition task, provides comparison feedback during vocal or instrumental training, or organizes a human–AI co-creation process into steps that learners can monitor, revise, and reflect on. In this sense, learner–task fit should be treated as a hypothesis-generating principle for future research, rather than as a firmly established rule.

### 5.3. Temporal Boundaries

The findings concerning intervention dose suggest that AI-assisted music learning may show different temporal patterns across outcome domains, although these patterns should not be interpreted as a precise dose–response function: motivation-related benefits appeared more responsive to AI exposure in the early stages of intervention, whereas higher-order cognition outcomes—such as SRL and creative performance tended to show larger estimates under longer intervention periods. Overall, this pattern is broadly consistent with prior work on the “novelty effect” in educational technology (Bognár & Khine, 2025; Jeno et al., 2019; Tsay et al., 2020), and also aligns with the present framework of “novelty decay and deep schema construction” (Clark, 1983; van Merriënboer & Sweller, 2005; Zimmerman, 2002). However, given the small number of long-term studies in the higher-order cognition domain and the presence of several large effect sizes, this temporal pattern should be understood as a provisional trend rather than as a stable developmental rule.

From an educational perspective, these temporal patterns may correspond to different forms of AI integration. Short-term AI activities, such as introductory workshops, trial lessons, or brief creative tasks, may be sufficient to generate novelty, interest, and perceived accessibility. By contrast, longer interventions, such as semester-based composition projects, sustained instrumental practice with AI feedback, or repeated human–AI creative cycles, may provide more opportunities for learners to internalize strategies, revise outputs, and engage in reflective regulation. In this sense, the temporal pattern is not only a statistical issue of intervention duration, but also a pedagogical issue of whether AI use is organized as a one-off motivational trigger or as a repeated learning cycle.

Importantly, although motivation showed an overall declining pattern over time, motivational effects did not disappear under long-term intervention conditions, but remained positive. This suggests that so-called novelty decay does not necessarily imply motivational failure. Rather, it may indicate that the stronger stimulus initially driven by novelty gradually weakens and gives way to a more stable, though relatively milder, motivational state. This interpretation is consistent with the findings of Tsay et al. (2020), who reported in a long-term online gamified learning system that although novelty effects were evident at the beginning, student engagement did not collapse once novelty faded, but could continue to be maintained under appropriate system support. In the context of AI-assisted music learning, this suggests that although the novelty bonus of AI may diminish, such mechanisms as immediate feedback, personalized support, and enhanced control may still sustain continued engagement. From this perspective, the present findings also refine earlier cautionary views on the novelty effect (Clark, 1983).

Accordingly, the temporal boundary suggested here is best understood as a difference in outcome sensitivity rather than as a simple “longer is better” principle. For motivation, AI may activate engagement in the early phase through novelty, immediacy of feedback, and perceived control. By contrast, for SRL, creative performance, and other higher-order cognition outcomes, longer exposure may provide more opportunities for reflection, strategy internalization, iterative revision, and generative integration, but the strength and stability of this pattern remain uncertain. In other words, the present review points to a possible dose × outcome-type relationship, while also indicating the need for longitudinal studies that compare different intervention durations within the same music learning context.

### 5.4. Mechanisms of AI Support

The qualitative synthesis of the 12 studies indicates that AI support in music learning does not operate as a simple accumulation of functions. Rather, it is mainly implemented through three pathways: evaluative feedback, generative support, and adaptive personalization. Music tasks have a distinctive ecological character, requiring learners to coordinate cognitive monitoring, affective expression, bodily adjustment, auditory–motor control, and aesthetic judgment. Therefore, the same AI function may carry different educational meanings across practice, performance, composition, and human–AI co-creation contexts. It is through such situated human–AI interaction that AI support may become associated with motivational activation, SRL-related experiences, and the expansion of creative exploration.

At the level of learner experience, the key contribution of the present study lies not in demonstrating that AI improves a single specific skill, but in revealing the underlying psychological mechanisms through which these gains are generated. Across the qualitative evidence, what appears to matter is not novelty per se, but whether novelty can be transformed into competence, control, and agency. This is consistent with the core proposition of self-determination theory, namely that short-term interest is more likely to develop into sustained engagement when the learning environment supports learners’ competence and autonomy. In the music learning domain, such autonomy support has likewise been shown to enhance practice quality and engagement by fostering more self-determined forms of motivation (Bonneville-Roussy & Evans, 2025; Ryan & Deci, 2020). However, in music learning, competence and control are not merely cognitive perceptions; they are often experienced through concrete musical action. For example, learners may hear improvements in pitch and rhythm, adjust bodily movement or vocal production, and compare AI feedback with their own auditory and expressive goals. This helps explain why evaluative-feedback AI may be particularly useful when it makes musical progress audible, visible, and actionable. At the same time, such feedback does not automatically translate into SRL; it may support self-regulatory processes only when learners actively use it for goal setting, process monitoring, strategy adjustment, and reflection.

At the same time, the common patterns and key differences identified in this study may be understood as two sides of the same AI-supported music learning ecology. The former captures a shared enabling mechanism of AI—namely, lowering entry barriers through cognitive offloading so as to activate participation, while preserving room for critical filtering so as to sustain deeper learning. The latter clarifies the context dependency of this mechanism, showing that it is reconfigured according to task goals, developmental stage, and AI role positioning. In other words, the underlying logic revealed here is that the educational effectiveness of AI depends less on technological complexity per se than on whether its role positioning appropriately matches task demands and the forms of cognitive processing required by learners. This is broadly consistent with prior work showing that personalized AI feedback is more likely to directly support learning motivation and learning outcomes, whereas in human–AI co-creation contexts, systems that preserve learner agency and aesthetic judgment are more likely to support creativity, participation, and reflective processing (Väkevä & Partti, 2025; W. Wang et al., 2026). Accordingly, the effectiveness of AI lies not in maximizing the number of functions it offers, but in whether it is embedded into a given context in an appropriate way.

Overall, feedback-based AI is better positioned as a tool for supporting embodied monitoring and reflective practice; generative AI is better positioned as a means of expanding materials for human aesthetic judgment; and adaptive AI should calibrate task demands without replacing learner agency. This also suggests that, in music learning and in arts and humanities education more broadly, educational outcomes are shaped not only by cognitive efficiency but also by embodiment, affective engagement, agency, and aesthetic judgment. Therefore, the most appropriate role of AI in music learning is not that of a universal substitute, but that of a supportive collaborator aligned with task goals, learner needs, and human judgment.

## 6. Limitations

Although this study used three-level meta-analysis and qualitative synthesis to provide a relatively systematic account of the effects, boundary conditions, and mechanisms of AI-assisted music learning, several limitations remain.

First, only English-language journal articles and conference papers were included, which means that relevant evidence published in other languages or in the grey literature may have been missed. It is nevertheless noteworthy that, even within English-language databases alone, empirical studies by Chinese scholars accounted for a substantial proportion of the included evidence, highlighting the high level of activity in this region in the application of AI to music education.

Second, because the aim of this study was to synthesize learner-outcome evidence in AI-assisted music learning, a relatively broad range of AI-assisted music learning contexts was included. Although this broad inclusion strategy allowed the review to capture a wider range of AI-assisted music learning practices, it also reduced the specificity of the pooled effect estimates. Therefore, the pooled effects should be interpreted as broad, context-sensitive evidence patterns rather than as precise estimates for any single type of music education.

Third, to reduce sparse data problems, SRL and creative performance were pragmatically combined as “higher-order cognition” in some analyses. However, SRL and creativity remain conceptually distinct constructs, with different theoretical foundations, measurement traditions, and possible developmental pathways. Therefore, findings for higher-order cognition should not be interpreted as evidence that SRL and creativity respond to AI support in the same way. Rather, they should be understood as pragmatic moderator-level evidence; future studies should examine SRL and creativity separately when a larger evidence base becomes available.

Fourth, in examining the interaction between prior ability and task cognitive complexity, some cross-cells—especially those involving low-complexity tasks within the higher-order cognition domain—contained very few studies. Relatedly, the binary coding of prior ability and task cognitive complexity may have reduced the sensitivity of the moderator analysis. Although this coding decision helped avoid sparse cells, it may have obscured nonlinear or threshold-like patterns at the medium level. Although this partly reflects the inherent tendencies of current educational design, it objectively constrained the strength of the statistical inference and left the findings at the level of directional indications rather than well-validated stable patterns. With respect to intervention dose, the extremely high overall heterogeneity indicates substantial variation across studies in intervention design and dose classification. Moreover, the long-term higher-order cognition subgroup contained relatively few studies and several exceptionally large effect sizes. Although sensitivity analyses generally preserved the direction of the long-term pattern, the magnitude of this effect remained unstable. These findings should therefore be interpreted as exploratory trend-based evidence rather than as a precise and stable dose–response function. Future studies should retain three-level or continuous coding when larger evidence bases become available, so that intermediate levels of task demand and learner expertise can be examined more precisely.

Lastly, although multiple robustness procedures were employed—including three-level meta-analysis, cluster-robust models, extreme-value treatment, the exclusion of rough effect sizes, and leave-one-study-out analyses—to reduce variation, unusually large effect sizes still tended to cluster in studies using self-developed measures, instruments highly aligned with the intervention, or perception-based components. Supplementary subgroup analyses were added for measurement tool type and AI support type, but these analyses remained exploratory because several subgroup cells contained few studies and SRL could not be meaningfully examined at the subgroup level. Although such measures are sensitive to proximal changes, they are also more likely to inflate standardized effect sizes because of restricted variance, common-method bias, and expectancy effects. Accordingly, the practical meaning of these findings should be interpreted with caution, particularly when distinguishing between effects derived from objective assessments and those derived from self-report measures. Future music-related research urgently needs to adopt more standardized and cross-contextually comparable objective instruments in order to provide more precise effect estimates.

## 7. Future Research and Conclusions

First, current evidence suggests that motivational benefits are more likely to emerge in the short term as a novelty-related gain, whereas benefits for higher-order cognition require longer-term accumulation. However, long-term studies remain scarce. Future systematic reviews and meta-analyses could therefore focus specifically on medium- and long-term AI interventions in education, with the aim of mapping more precisely the decay or growth curves of different learning outcomes and identifying optimal combinations of intervention dose and frequency for sustaining gains in higher-order cognition.

Second, because high-quality quantitative evidence on AI-supported music SRL is still limited, future reviews and empirical studies should treat SRL as a central focus. In particular, they should examine how AI exerts differentiated enabling effects across the micro-phases of SRL, such as goal setting in the forethought phase, strategic monitoring in the performance phase, and self-evaluation in the reflection phase.

Third, the qualitative findings of this study suggest that evaluative-feedback AI is more likely to support self-regulation and practice quality, generative-support AI is more likely to activate creative performance and creative expansion, and interactive/adaptive AI is more likely to enhance engagement, joint attention, and situated involvement. Accordingly, future research should move beyond simply comparing the presence versus absence of AI and instead examine more specifically the fit of different AI role positions—such as coach, mirror, creative partner, or collaborative other—across different task goals, age groups, and learner groups with different levels of prior ability. Further work should also investigate how these role positions influence learners’ sense of control, competence, agency, and aesthetic judgment.

Overall, although the differentiated enabling boundaries proposed in this study are not yet sufficient to capture the full range of music education contexts, the study makes an important contribution by beginning to address the evidential gap in the integration of deeper outcomes, the testing of boundary conditions, and the explanation of implementation mechanisms in AI-assisted music learning. More importantly, it provides an integrative framework for understanding how situated forms of AI may operate within the ecology of arts learning. Accordingly, the boundaries identified here should be understood as provisional rather than definitive, serving less as fixed conclusions than as an initial basis for subsequent refinement and validation across diverse learners, tasks, and instructional settings.

## Figures and Tables

**Table 1:** Interaction of task cognitive complexity and prior ability. *Note*: *k* = number of studies or effect sizes; *b* = regression coefficient; SE = standard error; CI = confidence interval.

Outcome Domain	*k* Studies	*k* Effects	Primary Three-Level Model	Supplementary Cluster-Robust Model	Interpretation
Motivation	13	28	*b* = −1.10, SE = 1.65, 95% CI [−4.34, 2.14], *p* = .506	*b* = −2.03, SE = 0.89, 95% CI [−3.77, −0.29], *p* = .022	Tentative; significant only in the supplementary robust model
Higher-order cognition	10	33	*b* = −0.12, SE = 3.11, 95% CI [−6.21, 5.97], *p* = .969	*b* = −0.72, SE = 0.83, 95% CI [−2.34, 0.91], *p* = .386	Not supported

**Table 2:** Meta-regression and descriptive evidence for dose-related patterns across outcome domains.

Indicator	Estimate/Pattern	*p* Value	Interpretation
Overall pooled effect	*g* = 1.33	—	Broad average across heterogeneous outcomes
Total heterogeneity	*I*^2^ = 96.8%	—	Very high heterogeneity
Long-term × higher-order cognition	*b* = 2.24	<.001	Higher-order cognition showed larger estimates under long-term interventions
Dose slope within higher-order cognition	*b* = 1.20	<.001	Positive dose-related trend
Dose slope within motivation	*b* = −0.07	.888	No clear dose-related increase
Motivation: short/medium/long	1.03/0.82/0.79	—	Motivation did not increase with longer dose
Higher-order cognition: short/medium/long	0.56/0.69/2.23	—	Larger estimates appeared in long-term interventions
Sensitivity analyses	Direction generally retained	—	Magnitude remained unstable due to small subgroup and large effects

## Data Availability

The original contributions presented in this study are included in the article/Supplementary Materials. Further inquiries can be directed to the corresponding author.

## Supplementary Materials

The following supporting information can be downloaded at https://www.mdpi.com/article/10.3390/jintelligence14070126/s1: Supplementary S1: Table S1: Database-specific search strategies; Table S2: Characteristics of Studies Included in the Meta-analysis; Table S3: Characteristics of Studies Included in the qualitative synthesis. Supplementary S2: Extracted effect-size classification table; Supplementary S3: Extracted effect sizes and outcome classification; Supplementary S4: Core meta-analysis RQ scripts.
