# Supplementary material for: Differentiated Empowerment and Boundary Effects of AI-Assisted Music Learning: A Mixed-Methods Study of Learning Motivation, Self-Regulated Learning, and Creative Performance
Source: J Intell. 2026 Jul 1;14(7):126. doi: 10.3390/jintelligence14070126 (PMC13413095; doi:10.3390/jintelligence14070126)
Supplement: Supplementary file 1 [file jintelligence-14-00126-s001.zip › Supplementary S1.pdf]

Table S1. Database-specific search strategies

Database	Search field	Search strategy
Scopus	TITLE-ABS-KEY	TITLE-ABS-KEY(("artificial intelligence" OR AI OR "machine learning" OR "deep learning" OR "neural network*" OR "generative AI" OR AIGC OR "intelligent tutoring" OR "automated feedback" OR "AI-assisted" OR "AI-supported" OR "adaptive learning systems") AND ((music* OR musical) AND (learn* OR educat* OR teach* OR train* OR instruct* OR pedagog*)) AND (("musical creativity" OR creativ* OR originality OR "creative performance" OR "creative self-efficacy" OR "perceived creative competence") OR (motivate* OR interest OR enjoyment OR "task value" OR "learning interest" OR "motivation to learn" OR flow) OR ("self-regulation" OR SRL OR metacognit* OR "goal setting" OR "self-efficacy" OR "self-monitor*" OR monitor* OR "effort regulation" OR "self-evaluat*" OR "self-reflect*" OR forethought OR "error detection" OR "self-observation")))
Web of Science Core Collection	Topic / TS	TS=(("artificial intelligence" OR AI OR "machine learning" OR "deep learning" OR "neural network*" OR "generative AI" OR AIGC OR "intelligent tutoring" OR "automated feedback" OR "AI-assisted" OR "AI-supported" OR "adaptive learning systems") AND ((music* OR musical) AND (learn* OR educat* OR teach* OR train* OR instruct* OR pedagog*)) AND (("musical creativity" OR creativ* OR originality OR "creative performance" OR "creative self-efficacy" OR "perceived creative competence") OR (motivate* OR interest OR enjoyment OR "task value" OR "learning interest" OR "motivation to learn" OR flow) OR ("self-regulation" OR SRL OR metacognit* OR "goal setting" OR "self-efficacy" OR "self-monitor*" OR monitor* OR "effort regulation" OR "self-evaluat*" OR "self-reflect*" OR forethought OR "error detection" OR "self-observation")))
ERIC	Peer	("artificial intelligence" OR AI OR "machine learning" OR "deep learning" OR "neural network*" OR "generative AI" OR AIGC OR "intelligent tutoring" OR "automated feedback" OR "AI-assisted" OR "AI-supported" OR "adaptive learning

	Review	systems") AND ((music* OR musical) AND (learn* OR educat* OR teach* OR train* OR instruct* OR pedagog*)) AND (("musical creativity" OR creativ* OR originality OR "creative performance" OR "creative self-efficacy" OR "perceived creative competence") OR (motivate* OR interest OR enjoyment OR "task value" OR "learning interest" OR "motivation to learn" OR flow) OR ("self-regulation" OR SRL OR metacognit* OR "goal setting" OR "self-efficacy" OR "self-monitor*" OR monitor* OR "effort regulation" OR "self-evaluat*" OR "self-reflect*" OR forethought OR "error detection" OR "self-observation"))
PsycINFO / PsycArticles	TI OR AB OR SU	TI(("artificial intelligence" OR AI OR "machine learning" OR "deep learning" OR "neural network*" OR "generative AI" OR AIGC OR "intelligent tutoring" OR "automated feedback" OR "AI-assisted" OR "AI-supported" OR "adaptive learning systems") AND ((music* OR musical) AND (learn* OR educat* OR teach* OR train* OR instruct* OR pedagog*)) AND (("musical creativity" OR creativ* OR originality OR "creative performance" OR "creative self-efficacy" OR "perceived creative competence") OR (motivate* OR interest OR enjoyment OR "task value" OR "learning interest" OR "motivation to learn" OR flow) OR ("self-regulation" OR SRL OR metacognit* OR "goal setting" OR "self-efficacy" OR "self-monitor*" OR monitor* OR "effort regulation" OR "self-evaluat*" OR "self-reflect*" OR forethought OR "error detection" OR "self-observation"))) OR AB((same search string)) OR SU((same search string))
RILM	TI OR AB OR SU	TI(("artificial intelligence" OR AI OR "machine learning" OR "deep learning" OR "neural network*" OR "generative AI" OR AIGC OR "intelligent tutoring" OR "automated feedback" OR "AI-assisted" OR "AI-supported" OR "adaptive learning systems") AND ((music* OR musical) AND (learn* OR educat* OR teach* OR train* OR instruct* OR pedagog*)) AND (("musical creativity" OR creativ* OR originality OR "creative performance" OR "creative self-efficacy" OR "perceived creative competence") OR (motivate* OR interest OR enjoyment OR "task value" OR "learning interest" OR "motivation to learn" OR flow) OR ("self-regulation" OR SRL OR metacognit* OR "goal setting" OR "self-efficacy" OR "self-monitor*" OR monitor* OR "effort regulation" OR "self-evaluat*" OR "self-reflect*" OR forethought OR "error detection" OR "self-

observation"))) OR AB((same search string)) OR SU((same search string))

Table S2. Characteristics of Studies Included in the Meta-analysis

**No.**	**Author(s), Year**	**Title**	**Study Design**	**Country**	**Intervention Duration Category**	**Prior Ability Level**	**Task Cognitive Complexity**	**Experimental Group**	**Control Group**
1	Li et al., 2025	AI-assisted feedback and reflection in vocal music training: effects on metacognition and singing performance	RCT	China	Short-term	Low Ability	Medium	38	42
2	Yin & Guo, 2025	An Artificial Intelligence-Based Interactive Learning Environment for Music Education in China: Traditional Chinese Music and Its Contemporary Development as a Way to Increase Cultural Capital	CCT	China	Short-term	Medium Ability	High	56	76
3	Zhang, 2025	Compositional tools based on artificial intelligence for choral artistic education: Enhancing creative skills in choral arrangements	RCT	China	Long-term	High Ability	High	35	35

No.	Author(s), Year	Title	Study Design	Country	Intervention Duration Category	Prior Ability Level	Task Cognitive Complexity	Experimental Group	Control Group
4	Xin, 2024	Diversified curriculum innovation of college music education under deep learning model	CCT	China	Medium-term	Low Ability	Medium	52	55
5	Yuan, 2024	Does AI-assisted creation of polyphonic music increase academic motivation?	RCT	China	Short-term	High Ability	High	49	49
6	Liu & Guo, 2025	Effectiveness of AI-Driven Vocal Art Tools in Enhancing Student Performance and Creativity	CCT	China	Medium-term	High Ability	High	79	79
7	Ou et al., 2025	Exploring the impact of AI-assisted practice applications on music learners' performance, self-efficacy, and self-regulated learning	Mix （CCT）	China	Long-term	High Ability	Medium	20	20
8	Wang & Zhang, 2025	Enhancing opera vocal education through advanced machine learning algorithms: analytics for talent development and curriculum design	CCT	China	Long-term	High Ability	High	60	58
9	Wang, 2025	Hybrid models of piano instruction: How combining traditional teaching methods with personalized AI feedback affects learners’ skill	RCT	China	Long-term	Low Ability	Low	40	59

No.	Author(s), Year	Title	Study Design	Country	Intervention Duration Category	Prior Ability Level	Task Cognitive Complexity	Experimental Group	Control Group
		acquisition, self-efficacy, and academic locus of control							
10	Gai, 2025	Implementation of an Innovative Approach to Vocal Training in College: The Case of Artificial Intelligence Technologies NSynth, Sing Like Me, and Flow Machines	CCT	China	Long-term	High Ability	High	220	55
11	Lv, 2023	Innovative music education: Using an AI-based flipped classroom	RCT	China	Long-term	Low Ability	Low	59	59
12	Liu & Liao, 2025	Integrating IBM Watson BEAT generative AI software into flute music learning: the impact of advanced AI tools on students' learning strategies	RCT	China	Medium-term	High Ability	High	112	108
13	Wang, 2025	Integration of AI GPTs in music education and their impact on students' perception and creativity	CCT	China	Medium-term	High Ability	Medium	283	283

No.	Author(s), Year	Title	Study Design	Country	Intervention Duration Category	Prior Ability Level	Task Cognitive Complexity	Experimental Group	Control Group
14	Liu, 2025	Modern AI program Chinese choral arts: cognitive training and motivation of college choristers	CCT	China	Short-term	Low Ability	High	30	30
15	Zhang & Li, 2025	Musical education and academic motivation in highly developed AI technology	CCT	China	Medium-term	High Ability	Low	110	110
16	Liu et al., 2022	National Ballad Creation Education Under Artificial Intelligence and Big Data	CCT	China	Medium-term	Low Ability	High	70	70
17	Xu & Xu, 2024	Psychological factors influencing successful music learning using deep learning technologies	CCT	China	Medium-term	Low Ability	High	118	120
18	Li & Wu, 2025	The Auxiliary Function and Realization Mechanism of Artificial Intelligence in Cross-Cultural Traditional Music Education	RCT	China	Medium-term	Low Ability	Medium	75	75
19	Tao, 2026	The Impact of AI-Based Educational Applications on University Students' Piano	RCT	China	Medium-term	High Ability	Medium	30	30

No.	Author(s), Year	Title	Study Design	Country	Intervention Duration Category	Prior Ability Level	Task Cognitive Complexity	Experimental Group	Control Group
		Skills and Self-Efficacy							
20	Zhuang & Li, 2025	The influence of collaborative music creation supported by generative artificial intelligence on students' creativity.	CCT	China	Long-term	Medium Ability	High	202	203
21	Kuldanov et al., 2025	The Use of Data-Based Emotion Recognition Systems for the Development of Emotional and Musical Creativity in Vocal Students	RCT	Kazakhstan	Medium-term	Medium Ability	High	64	64
22	Baxi et al., 2025	HYBRID AI-HUMAN MUSIC COMPOSITION FOR PEDAGOGY	Mix (CCT)	India/UAE	Medium-term	Medium Ability	High	22	21
23	Dash et al., 2025	SENTIMENT-BASED FEEDBACK IN ART EDUCATION	CCT	India/UAE	Medium-term	Low Ability	Low	34	34

Table S3. Characteristics of Studies Included in the qualitative synthesis.

No.	Study	Country	Age Group	AI Support Type and Music Task	Main outcomes	Implementation of support	Learner experience

1	Dong et al., 2024	United States	Children	Adaptive/interactive generative support; musical production, emotional expression, music and sound synthesis	Motivation, creative performance	Students acted as “directors” and used graphical programming to control a social robot’s movements and speech, collaboratively creating and performing a human–AI musical production across four modules: Acting, Dance, Music & Sound, and Drawing	Students felt excited, found the experience enjoyable, and showed high engagement; their interest in the robot developed from initial novelty into sustained exploratory enthusiasm. Teachers also observed high participation, although they were concerned about possible distraction
2	Lin et al., 2025	China	Adolescents	Evaluative feedback-based support; a MIDI platform for music theory, beat training, and play-along practice	Motivation	Through Follow mode, gesture tracking, MIDI comparison, and multimedia demonstrations, the system provided real-time visual cues and correctness verification, while learning progression was advanced according to pass rates	Students perceived the system as interesting, easy to use, and low-threshold, which enhanced conceptual understanding and learning initiative

3	Wani et al., 2025	India	Mixed-age learners	Generative support; form analysis, harmony generation, and composition	Motivation, SRL, creative performance	Students used tools such as AIVA, Amper, and MuseNet to generate melodies and harmonies, and then engaged in selection, combination, and further creative refinement. Teachers tracked the learning process through interviews, observations, and questionnaires	Students experienced the enjoyment of experimentation, collaboration, and expanded creative possibilities, while also feeling the need to constantly balance "algorithmic generation" and "human intuition"
4	Yao, 2025	China	Young adults	Generative support; music style exploration, music appreciation, and automatic composition	Motivation, creative performance	Teachers set the theme, students entered stylistic prompts, and the model automatically generated melodies and lyrics, which were then auditioned, compared, presented, and shared	Students felt that the threshold for creation was lowered and that the classroom became more active; they shifted from "listening" to "doing," with increased interest, attention, and verbal participation
5	Li, 2022	China	Children	Evaluative feedback/adaptive support; introductory piano learning, notation	Motivation	The intelligent piano supported children's play-along practice and error correction through waterfall notation, real-time capture, automatic	Children felt relaxed and happy, showed less resistance to piano practice, and became

				reading, rhythm, and error correction		tempo reduction, auditory prompts, and gamified lessons	more willing to participate proactively
6	Cui, 2023	China	Adults	Evaluative feedback-based support; AR piano learning, notation reading, action imitation, and independent practice	Motivation, SRL	Students used applications such as AR Pianist, Flowkey, and Simply Piano for independent practice through virtual performance demonstrations, slow playback, and real-time audiovisual feedback	Students felt that learning became easier to understand and more comfortable, and they were better able to correct errors independently from the teacher, thereby developing autonomous practice ability
7	Nakajima et al., 2026	Japan	Adolescents	Generative support; lyric generation, songwriting, and AI ethics	Motivation, SRL, creative performance	Through a “know → create → reflect/share” cycle, students first learned basic AI concepts and ethics, then used ChatGPT to generate lyrics and Suno to generate songs, while repeatedly revising prompts, selecting versions, and presenting their work	Students felt surprised and excited by the experience of turning text into music, and also developed an awareness of verifying AI outputs and avoiding blind trust. At the same time, some expressed

							concerns about dependence on AI or performance-related anxiety
8	Sun, 2025	China	Adults	Generative/personalized support; information searching, problem solving, and critical thinking	Motivation, SRL	Students used AI as a tool to complete music-related and academic tasks, supporting learning through active questioning, information verification, and problem solving	Students reported stronger feelings of control, self-efficacy, and confidence in handling complex tasks, and became more willing to engage proactively in learning
9	Ou et al., 2025	China	Mixed-age learners	Evaluative feedback-based support; intonation and rhythm scoring and error correction in violin practice	SRL, motivation	In daily practice, learners used Violy’s scores, error markings, and staged feedback to set goals, monitor progress, correct errors, and adjust through reflection	Learners felt that practice became more goal-directed; improvements in scores fostered confidence and a sense of achievement, while also reducing dependence on the

							teacher's immediate presence
10	Baxi et al., 2025	India	Young adults	Generative support; human–AI collaborative composition, melody generation, and harmonic arrangement	Motivation, SRL, creative performance	Students used AI as a brainstorming partner in a hybrid composition process of "AI suggestion → student evaluation/modification/rejection → further revision," and reflected on their creative work through logs and expert ratings	Students felt reduced blank-page anxiety and an expanded space for ideas, but also continuously experienced tension between human intuition and algorithmic suggestions, requiring critical listening and self-restraint
11	Roldan-Cardona et al., 2025	Colombia / Ecuador	Children	Adaptive/evaluative feedback-based support; preschool music concepts, rhythm, melodic creation, and interactive quizzes	Motivation	Children participated in multimodal interactive activities through tools such as Genially and Educaplay, learning musical and linguistic content through audiovisual stimuli and practice tasks	Children experienced the classroom as more interesting, dynamic, and engaging, with especially higher interest and participation among the 5–6-year-old group

12	Addessi & Pachet, 2005	Italy	Children	Adaptive/interactive generative support; improvisation and turn-taking human–AI performance	Motivation, creative performance, SRL	The Continuator analyzed children's playing style and, after a pause, continued generating melodies in a similar style, creating turn-taking improvisational interaction in "child–system" or "child–peer–system" formats	Children moved from surprise and excitement to rule-testing, focused attention, and active invention, showing a clear process of exploration, analysis, and creation
